# Dietary niche and the evolution of cranial morphology in birds

**DOI:** 10.1098/rspb.2018.2677

**Published:** 2019-02-20

**Authors:** Ryan N. Felice, Joseph A. Tobias, Alex L. Pigot, Anjali Goswami

**Affiliations:** 1 Department of Cell and Developmental Biology, University College London, London WC1E 6BT, UK; 2 Centre for Biodiversity and Environment Research, Department of Genetics, Evolution, and Environment, University College London, London WC1E 6BT, UK; 3 Department of Life Sciences, The Natural History Museum, London SW7 5DB, UK; 4 Department of Life Sciences, Imperial College London, Ascot, UK

**Keywords:** niche, macroevolution, diet, cranial morphology

## Abstract

Cranial morphology in birds is thought to be shaped by adaptive evolution for foraging performance. This understanding of ecomorphological evolution is supported by observations of avian island radiations, such as Darwin's finches, which display rapid evolution of skull shape in response to food resource availability and a strong fit between cranial phenotype and trophic ecology. However, a recent analysis of larger clades has suggested that diet is not necessarily a primary driver of cranial shape and that phylogeny and allometry are more significant factors in skull evolution. We use phenome-scale morphometric data across the breadth of extant bird diversity to test the influence of diet and foraging behaviour in shaping cranial evolution. We demonstrate that these trophic characters are significant but very weak predictors of cranial form at this scale. However, dietary groups exhibit significantly different rates of morphological evolution across multiple cranial regions. Granivores and nectarivores exhibit the highest rates of evolution in the face and cranial vault, whereas terrestrial carnivores evolve the slowest. The basisphenoid, occipital, and jaw joint regions have less extreme differences among dietary groups. These patterns demonstrate that dietary niche shapes the tempo and mode of phenotypic evolution in deep time, despite a weaker than expected form–function relationship across large clades.

## 1. Background

Observations of avian cranial evolution, and especially the beak, in response to ecology and behaviour are part of the bedrock of evolutionary theory. Beginning with Darwin's notes on the variety of beak morphologies among the finches on the Galapágos Islands [1], this system has been the textbook example of natural selection reinforcing a link among diversity, form, and function [2,3]. Skull morphology is highly variable and correlated with ecological and dietary factors across many other avian clades, especially in island radiations such as the honeycreepers of Hawai'i [4–6] and the vangas of Madagascar [7]. Galapágos finches are also one of the first examples of morphological evolution in response to extrinsic factors on short timescales [8,9]. These examples, in addition to non-avian radiations [10–15], support the widespread view that diversification is often the result of adaptive speciation that links morphology to behaviour, ecology, and diet. Together, these examples suggest that the skull and beak, as the food acquisition apparatus of birds, evolve to fit with the trophic niche of the lineage and the specific functional demands of diverse diets and foraging behaviours [3,4,16,17]. Whereas this view of the evolution of morphological disparity in birds is supported by several studies of postcrania [18–20], relatively few studies have tested whether cranial evolution is shaped by ecology on macroevolutionary scales.

A recent analysis of hundreds of bird species distributed across an elevation gradient in the Peruvian Andes demonstrated that cranial morphology is a strong predictor of dietary guild and foraging behaviour [21]. Conversely, studies focused on more restricted clades provide interesting counterexamples to this form–function link. For example, one recent study focusing on diurnal birds of prey (Falconidae, Cathartidea, and Accipitridae) demonstrated that diet does not predict beak shape [17]. Instead, phylogeny and allometry were found to be more important than trophic ecology in shaping cranial variation. However, raptorial birds primarily use talons for killing, rather than the beak and head. As such, this clade is less than ideal for addressing ecomorphological evolution in the cranium. In the more ecologically and behaviourally diverse Melphigidae (Australian honeyeaters), there is evidence that ecological niche partitioning is not associated with divergence in cranial morphology [22]. These earlier works have provided insight on the effects of diet of cranial morphology but are limited to focusing on restricted clades or regional avifaunas. Here, we expand the breadth of taxonomic sampling and quantification of skull shape to analyse how diet shapes morphology across modern birds.

Using a broad sample that encompasses extant avian diversity (159 of 195 extant families), we investigated the effects of diet and foraging behaviour on cranial morphology in light of recent evidence that the avian skull exhibits high modularity [23]. Analysis of the high-dimensional geometric morphometric (GMM) quantification of skull morphology has demonstrated the avian skull is composed of seven anatomical modules, each evolving with unique tempo and mode throughout the history of Neornithes [23]. The modular nature of the skull suggests that each cranial region is able to respond semi-independently to different selective pressures, with developmental complexity potentially influencing the evolvability of these regions [23]. We evaluated how cranial disparity and evolutionary rates are affected by trophic ecology, as summarized by two quantitative dimensions of the trophic niche: diet and foraging behaviour. Each cranial module is expected to have independent responses to selection for trophic niche. We predict that the rostrum and palate regions, composing the facial skeleton, have the strongest association with ecological traits. The cranial vault module, which contains attachments of jaw adductor musculature, and the occipital region, which contains the attachments of cervical musculature, are also expected to evolve in response to the biomechanical demands of various diets. Trophic ecology is predicted to influence not only the morphology of the skull but also the rate of morphological evolution. Typically, differences in rates of evolution among ecological niches are attributed to differences in the strength and pattern of selection in these groups [11,24,25]. This relationship has been demonstrated in a wide range of studies. For example, within sigmodontine rodents, insectivores evolve faster than omnivores and herbivores [25], whereas herbivores evolve faster than omnivores and carnivores in terapontid fish [11]. We tested whether similar patterns are present in birds by quantifying the relative rates of evolution among dietary groups in each cranial module.

## 2. Methods

### (a) Morphological data

Three-dimensional cranial morphology was quantified using a previously published dataset and procedure, composed of 352 species of extant birds (electronic supplementary material, table S1), representing nearly all living families [23]. Anatomical landmarks and semilandmark curves were placed on digital three-dimensional models of specimens, derived from surface and CT scans, using IDAV Landmark [26]. We then applied the semi-automated procedure in the Morpho (version 2.5.1) R package [27] to project surface semilandmarks from a template model on to each of the specimens, resulting in a total of 757 three-dimensional landmarks. Landmark data were subjected to a generalized Procrustes analysis, removing the effects of size, rotation, and position, using the geomorph (version 3.0.6) R package [28]. Landmarks were subdivided into seven anatomical modules (rostrum, palate, cranial vault, occipital, basisphenoid, pterygoid/quadrate, and naris) based on partitions supported in a previous analysis of the same dataset [23].

### (b) Phylogenetic hypothesis

**Figure 1.**
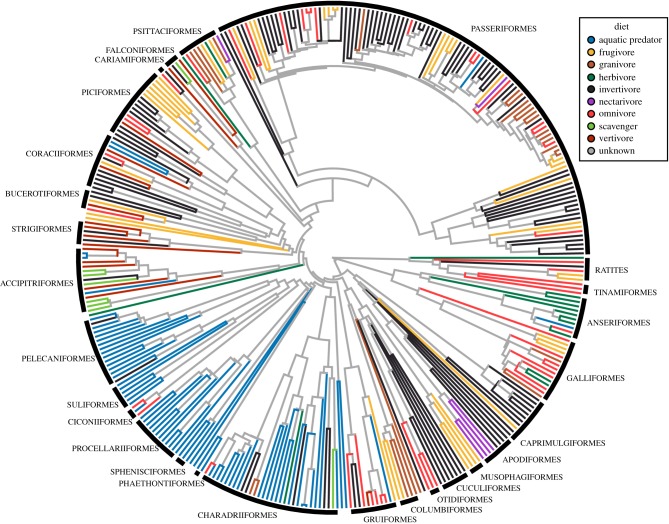
Phylogenetic hypothesis for sampled avian species (*n* = 352) with terminal branches coloured by dietary group. Each dietary group has multiple origins across the avian tree. Internal branches have unknown dietary niche. This time-calibrated tree was generated by grafting fine-scale branching patterns from ref. [29] on to the well-resolved backbone topology of ref. [31], following the procedures in ref. [32].

A composite phylogenetic tree was used for all phylogenetic comparative analyses (figure 1). First, a posterior distribution of 1000 trees was obtained from birdtree.org [29]. A single maximum clade credibility tree was then generated using TreeAnnotator [30]. The fine-scale relationships from this tree were grafted to a backbone tree from a recent genomic phylogeny [31] following published procedures [32]. We selected this backbone topology as it has been used in many recent studies of avian macroevolution [23,32,33] and because it represents a very well-supported hypothesis of the relationships among extant taxa, with a posterior probability of 1 for all but one node [31].

### (c) Ecological trait data

To quantify the trophic niche of each species, we estimated (i) the type of resources consumed (dietary group) and (ii) the foraging behaviours used to obtain these resources. We classified all species into dietary groups based on data from Wilman *et al*. [34] quantifying the dietary contribution of 10 different food categories (‘invertebrates’, ‘terrestrial vertebrates' (ectotherms, endotherms, or unknown), ‘fish’, ‘carrion’, ‘fruit’, ‘seeds’, ‘nectar’, and ‘other plant material’). Our final database was comprised of scores for nine resource types (terrestrial invertebrates, aquatic invertebrates, terrestrial vertebrates (hereafter, ‘vertebrates’), fish, carrion, fruit, seeds, nectar, and other plant material). Here, we modified these categories by combining all terrestrial vertebrate prey items into a single ‘vertebrate’ group and by scoring aquatic invertebrates (e.g. squid) with ‘fish’ (rather than ‘invertebrates’) to form an ‘aquatic animal’ and a ‘terrestrial invertebrate’ category. Following previous studies [21], we assigned species obtaining the majority (greater than or equal to 60%) of their resources from any one of these eight food categories to the corresponding dietary group, with the remaining species classified as ‘omnivores’ (*n* = 47). Thus, our final database was comprised of species membership for nine dietary groups. Dietary groups for each species are provided in electronic supplementary material, table S1. Each dietary category evolved multiple times within the present taxonomic sample (figure 1), providing the necessary framework to test whether the evolution of a specific trophic niche consistently drives the evolution of a common cranial phenotype.

In addition to dietary group, we scored trophic ecology by foraging behaviour. These foraging behaviours describe both the diet and the substrate or method of obtaining the food item. As such, this is not independent from dietary group as a measure of trophic ecology, but is a more fine-scale description of the resource and potentially a proxy for niche partitioning and function. For example, these differentiate between species that prey on invertebrates during flight, or by probing into crevices, or by walking on the ground. Each of these are expected to have different implications for trait evolution if birds that acquire similar prey in different ways experience different selective pressures on skull morphology. Following the method used by Wilman *et al*. [34] for classifying avian diets, we used a standardized protocol to translate qualitative descriptions of foraging behaviour [35] into semi-quantitative scores in a systematic way. Species were scored across 30 different foraging behaviours, described in electronic supplementary material, File S2. If a single foraging strategy was described this received a score of 10. Where multiple foraging strategies were mentioned, we used general terms describing their relative frequency as an initial guide (e.g. ‘mostly’ > 6, ‘sometimes’ = 2, occasionally = 1), adjusting these scores according to the remaining content of the description. If no indication on the relative use of different strategies was provided, categories listed earlier in the description were up-weighted relative to those listed at the end. The result is a multivariate description of foraging behaviour for each species.

### (d) Phylogenetic comparative methods

We evaluated the strength of covariation between diet and shape using distance-based regressions, also known as permutational or non-parametric MANOVA. Distance-based methods are suitable for high-dimensional data (i.e. more trait dimensions than observations) such as the phenome-scale morphometric data used here [36–38]. Because cranial morphology in this dataset has been shown to have significant phylogenetic signal [23], we employed the version of this test that incorporates phylogenetic covariance [37]. Using the diet category as the independent variable, we conducted separate regressions with the entire landmark configuration as the dependent variable and with each of the seven modules as the dependent variable. An additional regression was performed to test whether dietary groups exhibit significantly different cranial centroid size. Significance was evaluated in each regression using the random residual permutation procedure (RRPP, a method for computing *p*-values in regressions and ANOVAs that is implemented in the geomorph R package) with 10 000 iterations [39].

We compared the rate of evolution across diet groups using the *σ*_mult_ metric, which describes the multivariate rate under a Brownian motion model of evolution [40]. Briefly, this method calculates the rate of evolution from the sum of the squared Euclidean distances between the phylogenetically transformed trait values at the tips of the tree and the estimated ancestral state at the root of the tree. To compare rates among subgroups within a tree, Euclidean distances are calculated for all taxa on the full phylogeny, and the sum of the squares is calculated for each subgroup. Significance is then calculated by simulating data across the tree with a single rate and comparing observed and simulated rate ratios between groups (see electronic supplementary material, S3 and [40]).

Because foraging behaviour was quantified as a multivariate trait, a different analytical approach was used to evaluate the relationship between foraging behaviour and cranial shape. We employed a phylogenetic two-block partial least squares (PLS) [41]. This non-parametric test quantifies the strength and significance of the correlation between two multivariate datasets without the assumption that one is dependent on the other [42]. Significance for the PLS tests were evaluated using 10 000 RRPP interactions [41].

## 3. Results

There is a significant relationship between diet category and shape in each module (*p* < 0.001, table 1) except for the naris (*p* < 0.223). However, the goodness of fit is weak (*R*^2^ < 0.10, table 1), indicating that diet is a poor predictor of cranial morphology. This result suggests that variation across the entire skull is not primarily shaped by dietary factors at this scale of analysis. This relationship may be underestimated because our dietary categories are coarse, such that finer-scale associations between cranial morphology and diet are potentially overlooked in our analyses. We assessed this possibility by subdividing dietary categories into more finely partitioned behavioural strategies (see below). In addition, a weak link between skull shape and diet has previously been reported for diurnal raptors, where dietary niches appear to be partitioned by size, rather than cranial morphology [17]. To test this hypothesis, we calculated a phylogenetic np-MANOVA with centroid size as the response variable. As with whole skull and module shape, skull size is significantly but weakly correlated with dietary category (*R*^2^ = 0.06, *p* = 0.02), meaning that allometric effects are not likely to be overwhelming ecologically driven differences in skull shape.

**Table 1.** Results of phylogenetic non-parametric ANOVA of whole skull or module shape and whole skull centoid size against dietary group.

module	sum of squares	*R*^2^	*F*	*Z*	*p*-value
whole skull	0.014	0.074	3.439	5.488	0.001
rostrum	0.004	0.067	3.080	4.195	0.001
vault	0.004	0.087	4.091	5.499	0.001
basisphenoid	0.000	0.072	3.347	5.260	0.001
palate	0.004	0.077	3.577	5.017	0.001
pterygoid and quadrate	0.001	0.097	4.616	5.599	0.001
naris	0.000	0.028	1.218	0.647	0.223
occipital	0.001	0.068	3.120	4.624	0.001
centroid size of whole skull	40953	0.0563	2.558	1.962	0.02

**Figure 2.**
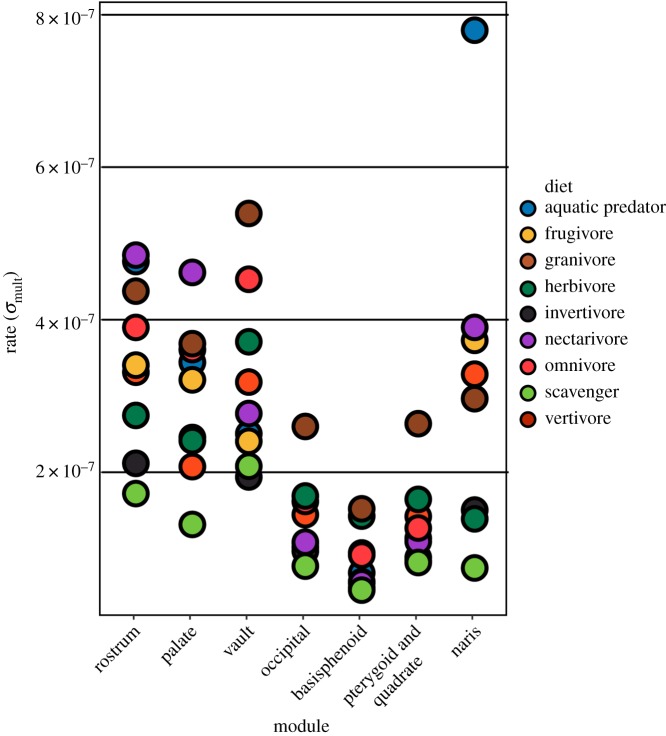
Evolutionary rates compared across dietary groups. Evolutionary rates (*σ*_mult_) were calculated for each module using the compare.evol.rates function in geomorph [40]. Rate values and results of significance tests are provided in detail in electronic supplementary material, S3.

Evolutionary rates are significantly different among dietary groups for all modules (figure 2; electronic supplementary material, table S3). Granivores are among the fastest evolving groups for modules except the naris, whereas terrestrial carnivores are among the slowest (figure 2). Herbivores exhibit fast-evolving basicranial features (basisphenoid, occipital) and cranial vault, but a slow-evolving palate and rostrum. Rates are similarly variable in nectarivores, which have rapid evolution in the rostrum, palate, and naris, but slow evolution in all other modules. Aquatic foragers have extremely high rates of evolution in the naris relative to other groups. This can be attributed to the loss of external nares in Sulidae (gannets and boobies) [43].

The heterogeneous rates of evolution among dietary groups could be caused by a variety of macroevolutionary factors. One explanation is that selection on cranial morphology is weak in diet groups with slow rates of phenotypic evolution (e.g. vertivores, invertivores), meaning that neutral processes (i.e. Brownian motion) would dominate phenotypic evolution in these groups. Alternatively, dietary groups with rapid trait evolution could have many adaptive optima, enabling rapid morphological shifts among peaks in the fitness landscape, compared to slow-evolving dietary groups with fewer peaks. Although methods do not currently exist to assess the likelihood of complex adaptive landscape models with high-dimensional data such as these [36], it is possible to gain some insight into these processes by examining morphospace occupation of each of the dietary groups. We conducted a principal components analysis (PCA) of the shape data in order to visualize morphospace occupation in each dietary group.

**Figure 3.**
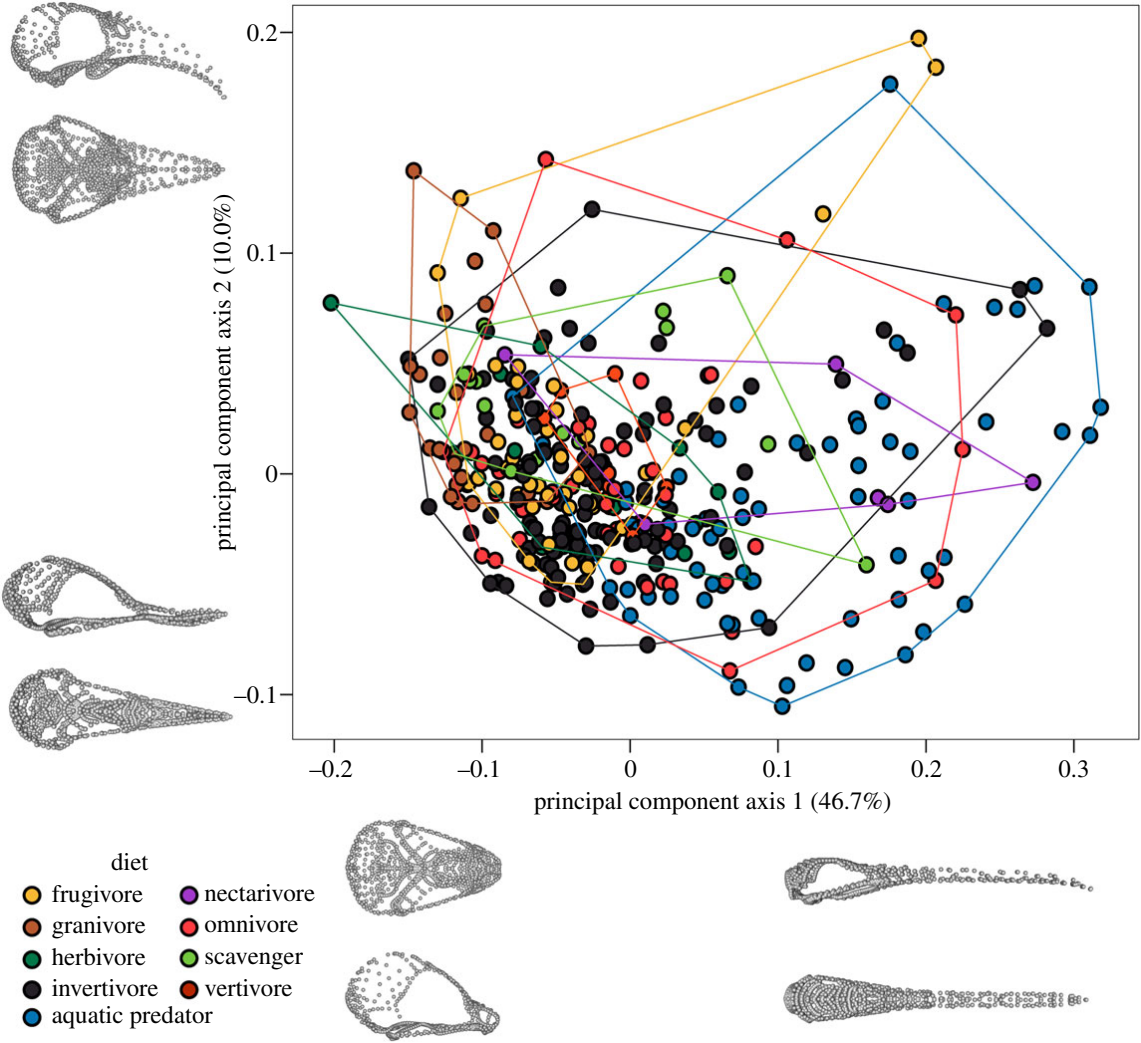
Principal component analysis of whole skull shape. Landmark configurations illustrate the shape change across principal component axis 1 (bottom) and axis 2 (left). PC axes 1 and 2 describe 46.7% and 10.0% of the overall shape variation, respectively. Landmark configurations were digitized and analysed on the right side only and are mirrored here to generate visualizations of the entire skull. (Online version in colour.)

As suggested by the low explanatory power of the np-MANOVA of diet on shape, there is broad overlap between dietary groups (figure 3). Omnivore, invertebrate, and aquatic dietary groups have the broad occupation of principal component (PC) axis 1, with all other diet groups occupying smaller regions of morphospace. Principal component axis 1 explains 46.7% of the total variance and primarily describes skull elongation. Taxa eating seeds and plants have short, robust beaks and are restricted to a region low on PC 1. They are distinct from nectarivores, which primarily have long beaks and score high on PC 1. The second PC axis, explaining 10% of total variance, describes dorsoventral beak curvature and mediolateral expansion of the palatine bones. The co-occurrence of narrow morphospace occupation and high rates of evolution in the granivore group suggests that there is repeated evolution of a small variety of seed-cracking phenotypes and that this ecology imposes strong constraints [44] and stronger selection. Like granivores, terrestrial carnivores inhabit a relatively restricted region of morphospace. The broad morphospace occupation of omnivores suggests that there are a broader range of viable phenotypes that fall into this behavioural category, as it is composed of a diversity of diet compositions and cranial functions. In frugivores, high cranial shape disparity is likely to be related to the diversity of fruit types and sizes, coupled with the coevolution between angiosperms and their avian seed dispersers [45,46].

**Figure 4.**
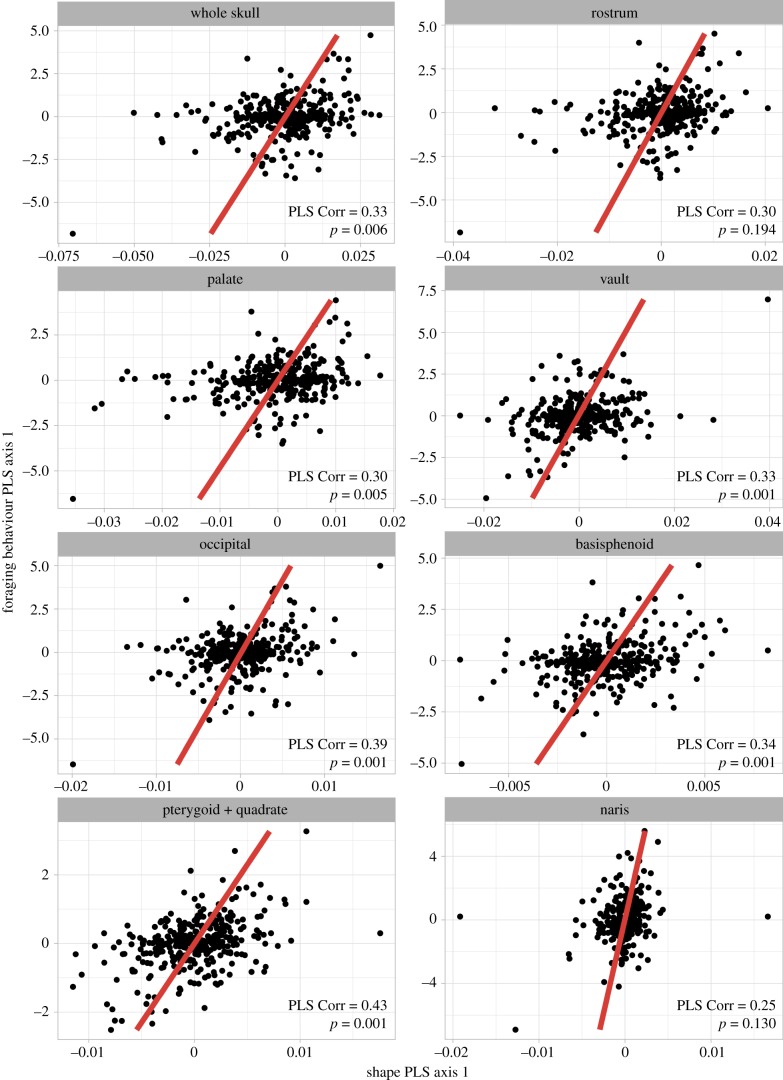
Correlations between skull shape and foraging behaviour. Fitted models are from phylogenetic two-block partial least-squares analysis. Foraging behaviour is quantified as the estimated proportion of food obtained via any of 30 foraging strategies within dietary niches, each defined by the substrate or manoeuvre involved (see electronic supplementary material, S2). (Online version in colour.)

Whereas diet category represents a coarse description of trophic niche, quantitative metrics of foraging behaviour have the potential to better describe resource use and thus serve as a more finely resolved proxy for function. As with diet, the shape of every module except for the naris has a significant but weak relationship with foraging behaviour (figure 4). The observed correlation between the first PLS axis ranges between 0.36 and 0.41 in these modules, with the first PLS axis explaining 24.5–33% of the covariation between foraging behaviour and shape in each module. The strongest PLS correlation is observed in the pterygoid and quadrate module. The first PLS axis for pterygoid and quadrate shape describes the relative size and orientation of the jaw articulation with respect to the pterygoid. Species with high PLS axis 1 scores have relatively large jaw articulations oriented at approximately 90° to the long axis of the pterygoid. Those with low PLS axis 1 scores have smaller jaw articulations oriented more in line with the pterygoid. The first PLS axis for foraging behaviour has high positive loading for the invertivore glean arboreal, vertivore glean arboreal, and vertivore glean ground categories. Foraging behaviours with high negative loading on this axis include ground and above-ground feeding granivores and foliavores. This indicates that in this region of the skull, morphology is weakly correlated with the relative importance of plant-based foraging relative to terrestrial, animal-based foraging (as opposed to aerial or aquatic animal foraging). Because this region includes the jaw articulation and contributes to cranial kinesis this might indicate the influence of the different biomechanical demands on the jaw joint across these foraging strategies. Although there are some outliers visible in the PLS plots, removing these data points and re-running PLS tests did not appreciably change the PLS correlation or significance values. This suggests that the reported results are not strongly influenced by individual outliers.

## 4. Discussion and conclusion

Diet category does not strongly predict cranial morphology in this broad sample. This disconnect between diet and skull shape, especially for the rostrum, contrasts with the patterns observed in adaptive radiations such as Darwin's finches that are known to exhibit correlated cranial morphology and trophic ecology [4,8,16]. This apparent contradiction can be partially attributed to the broad classification scheme with which we have defined diet. The underlying assumption of ecomorphological analyses such as this one is that taxa that share ecological traits are under similar selective pressures and these pressures drive the evolution of convergent morphologies. However, the functional demands, and thus selective pressures, experienced within each diet category are likely to be highly variable. For example, the ‘aquatic animals’ diet category contains both plunge-diving piscivores (boobies, pelicans) and terrestrial piscivores (herons, shoebill). Despite sharing a common trophic level, cranial structure and function is highly variable across these predatory taxa. As such, it may be unrealistic to predict that broad dietary categories such as the ones considered here, would have consistent cranial morphology.

Although diet is not strongly associated with cranial shape in this dataset, evolutionary rate and diet are certainly linked. Most strikingly, granivores have high rates of evolution in all cranial modules, whereas vertebrate-eaters evolve slowly. We hypothesize that these differences are related to the relative importance of the form-function link across diet groups. Seed-crushing granivores are highly dependent on biting performance to ensure foraging performance, fitness, and survival [3,16,47–49]. As such, in Darwin's finches, a number of cranial features are correlated with foraging strategy, including head width, beak aspect ratio, keratin thickness, and resistance to mechanical loading [16,47,48]. For this reason, granivore cranial morphology is expected to track diet closely. As these lineages evolve into new niches and exploit new food resources, cranial morphology is likely to rapidly evolve to fit with diet. We also recovered significantly high rates of evolution in the palate and rostrum in nectarivores. Nectarivores, like granivores, are expected to have high selection on cranial morphology, due to coevolution between beak and flower shape and size [50,51]. For these reasons, inferences about dietary ecology from fossil specimens should be considered carefully and multiple sources of evidence should be used, including phylogenetic and postcranial data.

By contrast, the low evolutionary rate in terrestrial carnivorous birds (raptors) may be due to relatively weak selective pressure on cranial morphology. Many carnivorous birds kill their prey with their talons, not their beaks, and many studies have demonstrated a significant relationship between foraging behaviour and hind limb morphology [52–54]. Moreover, the beaks of raptors all perform the same flesh-stripping role, regardless of prey type and size. Thus, the slow rates of cranial evolution observed in this dietary category may be a result of higher selective pressure in the postcranium than in the cranium.

These results point to the interesting conclusion that the clades that have been studied the most thoroughly in terms of adaptive cranial evolution form two ends of a spectrum. The prime examples of diet shaping cranial morphology in birds are from island radiations like Darwin's finches and Hawai'ian honeycreepers, which include a variety of seed-cracking and nectivorous specialists, both categories that show rapid evolution of cranial morphology in the present analysis. Recent research concluding beak morphology is shaped by non-dietary factors [17] happens to focus on carnivores, a dietary niche that shows slow cranial evolution. Thus, this discordance between analyses of adaptive evolution of cranial shape in these groups could be a product of diet-specific form–function associations and selective regimes.

Another explanation of the discordance between results on island radiations of birds and macroevolutionary studies is a matter of scale. In island radiations, cranial evolution has repeatedly been shown to be related to niche partitioning and resource use. However, on macroevolutionary scales, other factors may be more important. Expansion of cranial morphospace through evolutionary time can be attributed to the appearance of unique morphotypes at the origin of major clades. The differences among clade-specific morphologies (e.g. the distinctive bills of ducks, pelicans, parrots, and avocets) may be overwhelming the ecomorphological signal associated with ‘tinkering’ with these key phenotypes. This is consistent with the evolution of the beak (rhamphotheca) which was shaped first by early bursts of shape evolution (niche expansion) followed by fine-scale tuning of those morphologies (niche filling) [32].

Quantifying the importance of these one-off evolutionary innovations [55,56] and characterizing multivariate adaptive landscapes [36,57] remain major analytical hurdles in evolutionary biology. If different diets impose different selective regimes and modes of evolution on the evolution of the skull, it should eventually be possible to model these processes analytically. Until such tools are available, a path forward would involve comparative analysis on the strength of the link between form and function in the avian skull using functional morphology and biomechanics. Cranial function and its association with form has been quantified in only a small number of avian taxa using finite-element analysis [16,58] and three-dimensional modelling [59]. By expanding the taxonomic and ecological breadth of these studies, it will be possible to determine the extent to which foraging performance is a more important selective pressure in some dietary niches than in others.

Diet and foraging behaviour are significant predictors of cranial morphology, although the predictive power of this relationship is relatively weak at this broad scale of inquiry. Our results also highlight the significant differences in evolutionary rates among dietary groups, thus demonstrating how dietary ecology can influence phenotypic macroevolution. In the light of the present dataset and other recent large-scale analyses of craniofacial evolution [17,23,32], a clearer picture of the morphological diversification of birds is emerging. The evolution of the avian skull is constrained by complex interactions among intrinsic and extrinsic factors, including trait integration, cranial function, phylogenetic history, and ecological opportunity. Together, these factors result in complex, ever-changing adaptive landscapes. Further research into form–function relationships in the skull and evolutionary tempo and mode will begin to decipher the role that dietary diversity and adaptation have played in avian macroevolution.

## Supplementary Material

Ecological Trait Data

## Supplementary Material

Dietary Guild Descriptions

## Supplementary Material

Evolutionary Rates Analysis: Significance Testing Results

## Data accessibility

Surface scan data are available for download at www.phenome10k.org.
