# Supplementary material for: Dietary niche and the evolution of cranial morphology in birds
Source: Proc Biol Sci. 2019 Feb 20;286(1897):20182677. doi: 10.1098/rspb.2018.2677 (PMC6408879; doi:10.1098/rspb.2018.2677)
Supplement: Dietary Guild Descriptions [file rspb20182677supp2.pdf]

Supplemental Data File 3. Felice, Tobias, Pigot and Goswami. Dietary Niche and the Evolution of Cranial Morphology in Birds. Proc. R. Soc. B. doi:10.1098/rspb.2018.2677

Evolutionary Rates Analysis: Significance Testing Results

Evolutionary rates were compared among dietary guilds using the σ_{mult} statistic [38]. Signficance testing for this method is conducted via pairwise comparisons (i.e., does group A evolve and a significantly different rate than group B). This is achieved by simulating data on the phylogeny with a single rate of evolution for all groups and calculating ratios between evolutionary rates of groups, then iterating this process to produce a distribution of rate ratios. If the ratio of evolutionary rates between two groups is greater than 95% of the simulated ratios, the difference in rates between those groups is considered significant [38]. Figure A illustrates this procedure for three pairwise comparisons using the rostrum region data. The histogram represents the rate ratios generated by 10,000 iterations of the permutation procedure. Arrow "A" indicates the observed ratio between the rate of rostrum evolution in granivores and in nectarivores (ratio = 1.1). This observed ratio falls into the simulated distribution and is not statistically significant (p = 0.58, Table S3.a). Arrow "B" indicates the ratio observed between granivores and vertivores (ratio = 2.54, p = 0.0001), whereas arrow "C" indicates the observed ratio between nectarivores and vertivores (ratio = 2.82, p = 0.0001). Granivores and nectarivores both have significantly higher rates of rostrum evolution than vertivores, but granivores and nectarivores are not significantly different from one another. For each module there are 36 pairwise comparisons, for a total of 252 pair wise comparisons. We present the p-values of the pairwise comparisons in table form below, rather than illustrating all 252 visually. The observed evolutionary rates for each dietary guild and each cranial module are presented in Table A below and Figure 1.

Figure A: Example significance test: evolutionary rate comparisons in the rostrum

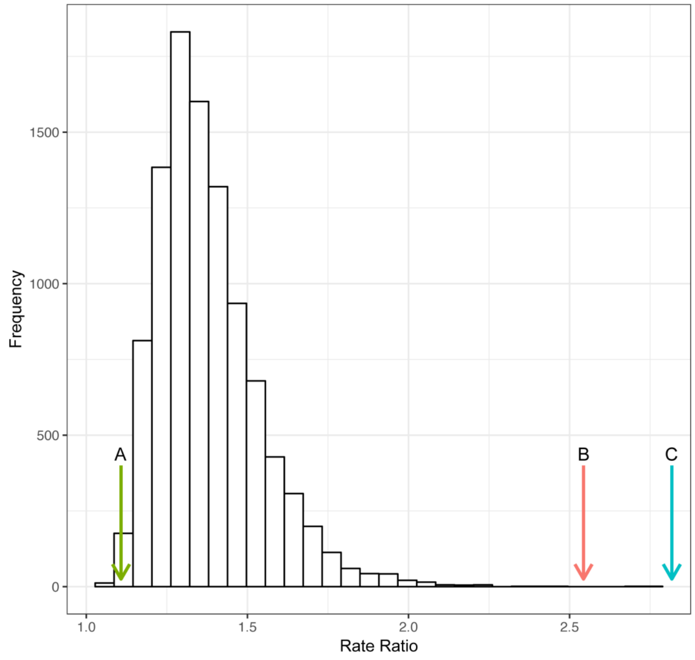

Table A: Observed Evolutionary Rates (σ_{mult})

	Rostrum	Vault	Basisphenoid	Palate	Pterygoid + Quadrate	Naris	Occipital
Aquatic Animals	4.76E-07	2.51E-07	6.81E-08	3.44E-07	1.13E-07	7.80E-07	1.02E-07
Carrion	3.31E-07	3.18E-07	9.41E-08	2.07E-07	1.42E-07	3.29E-07	1.44E-07
Fruit	3.40E-07	2.41E-07	5.65E-08	3.21E-07	8.80E-08	3.73E-07	1.07E-07
Invertebrates	2.12E-07	1.94E-07	4.83E-08	2.46E-07	8.37E-08	1.50E-07	9.65E-08
Nectar	4.85E-07	2.77E-07	5.49E-08	4.62E-07	1.09E-07	3.90E-07	1.08E-07
Omnivore	3.90E-07	4.53E-07	9.13E-08	3.61E-07	1.27E-07	2.97E-07	1.62E-07
Plants	2.74E-07	3.71E-07	1.42E-07	2.41E-07	1.64E-07	1.39E-07	1.69E-07
Seeds	4.38E-07	5.39E-07	1.52E-07	3.69E-07	2.64E-07	2.96E-07	2.60E-07
Vertebrates	1.72E-07	2.08E-07	4.54E-08	1.31E-07	8.16E-08	7.44E-08	7.69E-08

Tables B-H: P-values for pairwise comparisons of the evolutionary rates of dietary groups for each module. Shaded cells indicate dietary groups with significantly different rates of evolution.

Table B: Rostrum

	Aquatic Animals	Carrion	Fruit	Invertebrates	Nectar	Omnivore	Plants	Seeds
Carrion	2.18E-02							
Fruit	1.00E-04	8.71E-01						
Invertebrates	1.00E-04	4.60E-03	1.00E-04					
Nectar	9.24E-01	9.77E-02	5.59E-02	2.00E-04				
Omnivore	1.35E-02	3.23E-01	1.37E-01	1.00E-04	2.35E-01			
Plants	1.00E-04	3.20E-01	9.85E-02	2.90E-02	5.60E-03	5.80E-03		
Seeds	3.93E-01	1.12E-01	1.85E-02	1.00E-04	5.87E-01	2.77E-01	1.10E-03	
Vertebrates	1.00E-04	1.00E-04	1.00E-04	7.13E-02	1.00E-04	1.00E-04	1.90E-03	1.00E-04

Table C: Palate

	Aquatic Animals	Carrion	Fruit	Invertebrates	Nectar	Omnivore	Plants	Seeds
Carrion	1.90E-03							
Fruit	4.19E-01	8.20E-03						
Invertebrates	1.00E-04	2.98E-01	7.00E-04					
Nectar	1.11E-01	1.00E-03	5.57E-02	7.00E-04				
Omnivore	5.64E-01	1.20E-03	2.15E-01	1.00E-04	1.94E-01			
Plants	4.40E-03	4.36E-01	3.11E-02	8.89E-01	2.40E-03	2.50E-03		
Seeds	4.96E-01	1.80E-03	2.10E-01	1.00E-04	2.56E-01	8.41E-01	3.20E-03	
Vertebrates	1.00E-04	1.66E-02	1.00E-04	1.00E-04	1.00E-04	1.00E-04	3.00E-04	1.00E-04

Table D: Vault

	Aquatic Animals	Carrion	Fruit	Invertebrates	Nectar	Omnivore	Plants	Seeds
Carrion	2.30E-01							
Fruit	6.87E-01	1.69E-01						
Invertebrates	2.00E-03	9.80E-03	2.37E-02					
Nectar	6.60E-01	6.27E-01	5.38E-01	1.01E-01				
Omnivore	1.00E-04	7.99E-02	1.00E-04	1.00E-04	2.73E-02			
Plants	8.10E-03	5.10E-01	6.70E-03	1.00E-04	2.46E-01	2.06E-01		
Seeds	1.00E-04	1.39E-02	1.00E-04	1.00E-04	4.50E-03	1.79E-01	3.09E-02	
Vertebrates	2.07E-01	6.13E-02	3.48E-01	6.21E-01	2.54E-01	1.00E-04	2.50E-03	1.00E-04

Table E: Occipital

	Aquatic Animals	Carrion	Fruit	Invertebrates	Nectar	Omnivore	Plants	Seeds
Carrion	8.80E-02							
Fruit	6.22E-01	1.57E-01						
Invertebrates	5.27E-01	4.18E-02	2.90E-01					
Nectar	8.02E-01	3.25E-01	9.76E-01	6.27E-01				
Omnivore	1.00E-04	5.97E-01	6.00E-04	1.00E-04	8.35E-02			
Plants	1.60E-03	5.14E-01	5.80E-03	2.00E-04	8.53E-02	7.88E-01		
Seeds	1.00E-04	6.70E-03	1.00E-04	1.00E-04	2.00E-04	2.00E-04	1.45E-02	
Vertebrates	6.91E-02	8.70E-03	4.38E-02	1.29E-01	1.94E-01	1.00E-04	4.00E-04	1.00E-04

Table F: Basisphenoid

	Aquatic Animals	Carrion	Fruit	Invertebrates	Nectar	Omnivore	Plants	Seeds
Carrion	8.03E-02							
Fruit	5.06E-02	6.30E-03						
Invertebrates	1.00E-04	3.00E-04	7.27E-02					
Nectar	2.95E-01	3.97E-02	8.80E-01	5.24E-01				
Omnivore	2.10E-03	8.74E-01	1.00E-04	1.00E-04	1.44E-02			
Plants	1.00E-04	5.23E-02	1.00E-04	1.00E-04	1.00E-04	2.20E-03		
Seeds	1.00E-04	1.75E-02	1.00E-04	1.00E-04	1.00E-04	1.00E-04	6.83E-01	
Vertebrates	1.80E-03	7.00E-04	1.28E-01	6.36E-01	4.18E-01	1.00E-04	1.00E-04	1.00E-04

Table G: Pterygoid and Quadrate

	Aquatic Animals	Carrion	Fruit	Invertebrates	Nectar	Omnivore	Plants	Seeds
Carrion	3.31E-01							
Fruit	3.53E-02	4.31E-02						
Invertebrates	1.30E-03	1.74E-02	6.59E-01					
Nectar	8.85E-01	4.32E-01	4.23E-01	3.12E-01				
Omnivore	3.34E-01	6.42E-01	5.60E-03	1.00E-04	5.75E-01			
Plants	3.65E-02	5.86E-01	5.00E-04	2.00E-04	1.73E-01	1.63E-01		
Seeds	1.00E-04	1.14E-02	1.00E-04	1.00E-04	1.70E-03	1.00E-04	1.61E-02	
Vertebrates	5.50E-02	4.02E-02	6.71E-01	8.71E-01	3.24E-01	1.63E-02	1.60E-03	1.00E-04

Table H: Naris

	Aquatic Animals	Carrion	Fruit	Invertebrates	Nectar	Omnivore	Plants	Seeds
Carrion	3.00E-04							
Fruit	1.00E-04	5.85E-01						
Invertebrates	1.00E-04	4.00E-04	1.00E-04					
Nectar	4.90E-03	5.87E-01	8.63E-01	1.00E-04				
Omnivore	1.00E-04	6.56E-01	7.58E-02	1.00E-04	2.88E-01			
Plants	1.00E-04	1.30E-03	1.00E-04	6.28E-01	5.00E-04	1.00E-04		
Seeds	1.00E-04	6.77E-01	1.24E-01	1.00E-04	3.07E-01	9.92E-01	3.00E-04	
Vertebrates	1.00E-04	1.00E-04	1.00E-04	1.00E-04	1.00E-04	1.00E-04	3.70E-03	1.00E-04
