# Supplementary material for: Dietary niche and the evolution of cranial morphology in birds
Source: Proc Biol Sci. 2019 Feb 20;286(1897):20182677. doi: 10.1098/rspb.2018.2677 (PMC6408879; doi:10.1098/rspb.2018.2677)
Supplement: Evolutionary Rates Analysis: Significance Testing Results [file rspb20182677supp3.pdf]

Supplemental Data File 2. Felice, Tobias, Pigot and Goswami. Dietary Niche and the Evolution of Cranial Morphology in Birds. Proc. R. Soc. B. doi:10.1098/rspb.2018.2677

Dietary guilds

1. **Invertivore** (In) - invertebrates in air or terrestrial habitats.
2. **Aquatic predator** (Aq) - invertebrates and vertebrates in aquatic habitats.
3. **Frugivore** (Fr) - fruit.
4. **Nectarivore** (Ne) - nectar and plant exudates.
5. **Granivore** (Gr) - seeds.
6. **Herbivore** (Pl) - vegetation (not including seeds, nectar or fruit).
7. **Vertivore** (Vt) - terrestrial vertebrates.
8. **Scavenger** (Sc) - carrion and human waste.

Foraging guilds

1. **Invertivore aerial screen (IASC)** – species capturing flying invertebrates on the wing (e.g. swallows, swifts). Often described as 'hawking'. In contrast to ISA, IASC is characterized by continuous and extended flight with multiple items captured before landing.
2. **Invertivore aerial sally (ISA)** – species capturing flying invertebrates in mid-air, with the attack starting from a perch (i.e. branch, rock, fence post, telegraph wire, etc.) and then returning to a perch (e.g. jacamars, kingbirds, etc). 'Hawking' will sometimes refer to this category, but the key distinguishing feature is that only a single prey item is captured before returning to a perch.
3. **Invertivore sally to substrate (ISS)** – species capturing invertebrates (including arachnids, worms, molluscs, etc.) attached to the substrate (e.g. leaves, twigs, branches, rock faces, etc) following an aerial attack manoeuvre (e.g. flight, pounce, jump, hover).
4. **Invertivore sally to ground (ISG)** – species capturing invertebrates on the ground following an aerial attack manoeuvre (e.g. flying, gliding, dropping or pouncing) (e.g. chats, shrikes, kiskadee etc). The aerial manoeuvre may be followed by brief hopping toward prey (e.g. terns).
5. **Invertivore glean arboreal (IGA)** – species capturing invertebrates attached to the substrate (e.g. leaves, twigs, branches, grass, bamboo, stems, hanging dead-leaves [not dead leaves on the ground] etc). No aerial attack manoeuvre is involved.
6. **Invertivore glean bark (IGB)** – species capturing invertebrates attached to or concealed within large branches and trunks of trees (e.g. woodpeckers, treecreepers, woodcreepers, wallcreepers, nuthatches, sitellas, nuthatch vanga, etc.). This is distinguished from IGA by at least one criteria. First, the species employs specialized methods for moving over surfaces which are often, but not always, vertical and too large to be gripped by the closed foot (including creeping, climbing or scaling). Second, the species extracts prey from in/under the bark using specialized methods (including hammering, probing or chiselling). Also includes species capturing insects from rock and cliff-faces (though not just on boulders [see IGG]), habitually perching on or clinging to large mammals and species that feed on honey and beeswax (e.g honeyguides).
7. **Invertivore glean ground (IGG)** – species capturing invertebrates on the ground. In contrast to ISG, the search and attack manoeuvres take place on the ground (e.g. Thrushes). This includes species standing on the ground and gleaning insects from vegetation (e.g. tinamous or larks) but excludes species that jump or sally upwards to

capture prey from vegetation (ISS) or the air (ISA). The ground is dry and thus excludes aquatic habitats (including beaches, estuaries, wetlands and marshes [see AQGR]).

8. **Aquatic predator ground (AQGR)** – species capturing invertebrates or vertebrates while standing in aquatic habitats (including beaches, estuaries, wetlands and marshes) (e.g. storks, herons, shorebirds). Prey may be captured on the ground or on/under water. This category includes species capturing aquatic prey (e.g. fish) or terrestrial prey (e.g. grasshopper) in aquatic habitats.

9. **Aquatic predator perch (AQPE)** – species capturing invertebrates or vertebrates on/under water following a direct attack flight from a perch (e.g. some kingfishers).

10. **Aquatic predator air (AQAI)** - species capturing invertebrates or vertebrates on/under water during continuous flight (including dipping, hovering, pattering, snatching). In contrast to AQPE, prey item is identified while flying (not from perch). The predators body may partially submerge but does not plunge beneath the surface (AQPL). Includes kleptoparasitic species capturing fish by chasing other piscivores and forcing them to regurgitate (e.g. skuas, frigatebirds).

11. **Aquatic predator plunge (AQPL)** - species capturing invertebrates or vertebrates by plunging under water following continuous flight. The predators body submerges entirely beneath the surface, with the prey captured either by the momentum of the plunge or following propelled swimming.

12. **Aquatic predator surface (AQSU)** - species capturing invertebrates or vertebrates on/under water whilst swimming on the water's surface. In contrast to AQPE or AQAI there is no direct attack flight. The species may dip under the water but, in contrast to AQDI, contact with the surface is maintained.

13. **Aquatic predator dive (AQDI)** - species capturing invertebrates or vertebrates under water by diving from the surface (not the air, see AQPE and AQPL).

14. **Frugivore aerial (FAE)** – species foraging on fruits in flight, including those that hover to pluck fruit from bushes and trees (e.g. oilbird, some manakins).

15. **Frugivore glean (FGL)** – species foraging on fruits while perched (not in flight) above ground and plucking fruits from vegetation (e.g. toucans, hornbills).

16. **Frugivore ground (FGR)** – species foraging on fruits lying on the ground (e.g. thrushes, trumpeters).

17. **Nectarivore aerial (NAE)** – species feeding on nectar or other plant exudates (e.g. sap) while in flight (e.g. hummingbirds).

18. **Nectarivore glean (NGL)** – species feeding on nectar or other plant exudates (e.g. sap) while perched, including nectar predators that pierce corollas (e.g. sunbirds, flowerpiercers). Species feeding on honey (e.g. honeyguides) are included under IGB.

19. **Granivore above-ground (GRA)** – species foraging on seeds, grains and nuts taken from vegetation (e.g. trees, grass stems) while perched (e.g. seedeaters, finches).

20. **Granivore ground (GRG)** – species foraging on fallen seeds, grains and nuts collected from the ground. Including birds that eat grains by, on, and under shallow water (e.g. partridges, pheasants, finches).

21. **Herbivore above-ground (PA)**– species foraging on leaves, buds, blossom, or other vegetation (except fruit and nectar). The food is taken from above ground, generally while the species is perching on branches or other stems. Generally, a small part of diet, except for the Hoatzin.

22. **Herbivore ground (PG)** – species foraging on leaves, buds, blossom, or other vegetation (except fruit) taken while the species is on the ground. The vegetation may itself be off the ground, so long as the species forages terrestrially.

23. **Herbivore aquatic (PAQ)** – species foraging on aquatic vegetation either below or above the water surface (algae, pondweed, waterside vegetation). The species collects vegetation while under water, sitting on the water's surface, or wading.
24. **Vertivore aerial screening (VASC)** – species capturing vertebrate prey during flight. Both predator and prey are in flight (e.g. some falcons)<br>25. **Vertivore aerial to substrate (VAS)** – species capturing prey on branches or the ground by diving from the air, usually after circling, hovering or patrolling/quartering in flight (e.g. kestrels, kites,harriers, some owls).<br>26. **Vertivore sally to substrate (VSS)** – species capturing prey on branches or the ground by diving from a perch (e.g. many owls, eagles).<br>27. **Vertivore glean ground (VGG)** – species capturing prey on the ground, including eggs in ground nests, while they themselves are also walking or running on the ground (e.g. secretary bird, seriemas, ground hornbills).<br>28. **Vertivore glean arboreal (VGA)** – species capturing prey from foliage, branches, epiphytes, cavities, bark or other arboreal substrate while perched on the substrate. There is no flight attack involved. This includes eating bird chicks from arboreal nests and drinking blood while perched on mammals (e.g. oxpeckers).
29. **Scavenger aquatic (SCA)** – species eating carrion (dead animal or fish remains) from water (e.g. petrels, albatrosses).<br>30. **Scavenger ground (SCG)** – species eating carrion (dead animal or fish remains) on the ground (e.g. vultures).
